# Bridging the gaps for women in substance use treatment

**DOI:** 10.3389/fpsyt.2025.1700502

**Published:** 2026-01-02

**Authors:** Nancy Poole

**Affiliations:** Centre of Excellence for Women’s Health, Vancouver, BC, Canada

**Keywords:** substance use treatment, women, gender-specific, trauma-informed, equity

## Abstract

This perspective provides an overview of a rapid review of research, and discussions held with Canadian treatment providers, that together illustrate how gaps persist in treatment and support for women with substance use concerns; and how attention to four elements of gender-specific treatment for women could ameliorate the situation.

## 1 Introduction

In 2004, the United Nations Office on Drugs and Crime (UNODC) released the *Substance Use Treatment and Care for Women* report, which captured case studies and lessons learned in efforts to develop and improve substance use treatment globally. In this report, the authors defined “gender responsive” substance use treatment programs as those that considered the needs of women in all aspects of their design and delivery (1). Guiding principles for gender responsive treatment included: creating supportive environments; initiating relational practices and policies; integrating mental health, substance use and trauma/violence services; improving the social determinants of health; and creating community based comprehensive care (1).

Over twenty years later, action is still needed on gender responsive treatment, that addresses both the sex-related (biological) and gender-related (social) factors affecting the health and wellbeing of women who use substances, and their children. Such action is now critical, as the prevalence of substance use by women and girls continues to increase (2–4), and there is increased evidence of the harms of substance use on women’s health (5, 6).

In 2024, the Centre of Excellence for Women’s Health invited Canadian researchers and service providers interested in women’s substance use treatment and recovery to three virtual meetings to discuss research and service delivery priorities for the advancement of gender specific treatment and recovery. In addition, we conducted a rapid review of best practices in sex- and gender-informed treatment and recovery for women and girls, and an environmental scan of gender-specific treatment and recovery programs for women across the country.

This perspective article provides an overview of the research and discussions, that illustrate how gaps persist in treatment and support for women with substance use concerns, and how attention to four elements of gender-specific treatment for women could ameliorate the situation.

## 2 Women’s substance use treatment requiring action

**Figure 1:**
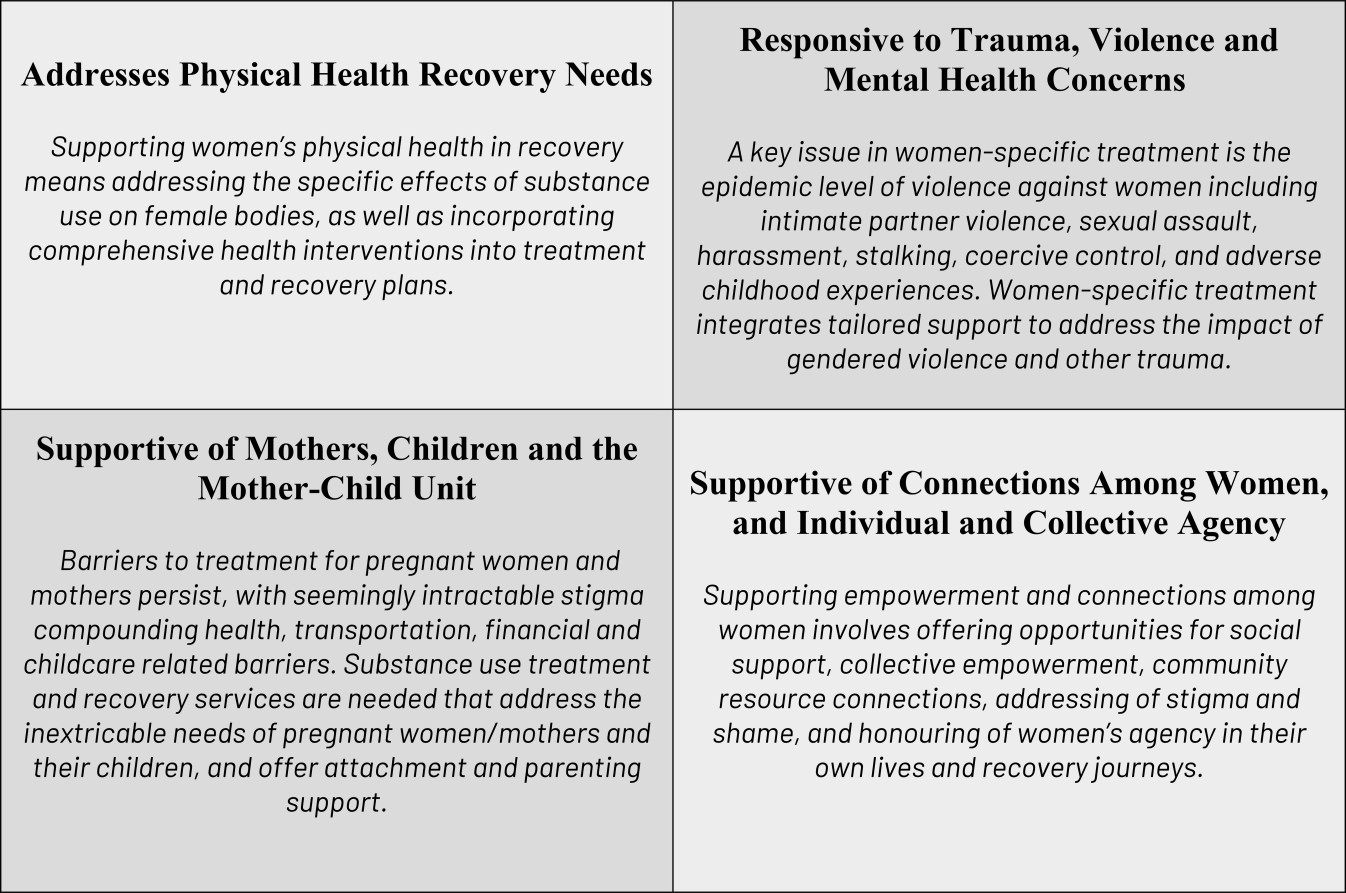
Four elements of women’s substance use treatment and recovery.

Canadian treatment providers identified four elements of women’s substance use treatment (see **Figure 1**) requiring further attention and action. Underlying these four elements are social and structural determinants of health, and Indigenous cultural wellness principles of generating a sense of purpose and belonging, and introducing meaning and hope to women who are using substances (7, 8).

### 2.1 Addresses physical health needs

Women constitute a vulnerable population in need of tailored substance use treatment and recovery support because women in treatment often have sex- and gender-specific co-occurring physical illnesses and health conditions (9, 10), such as liver, kidney, gastrointestinal, and heart diseases; difficulties with ovulation and menstruation; cancers; increased risk of sexual victimization; and anxiety, depressive, posttraumatic stress, and related health issues (5, 10–13). Women in treatment also report higher rates of chronic pain and are prescribed, and rely on, opioid medications more often than men (14). Thus, in treatment settings it is important to provide education and programming that is responsive to the impacts of substance use on female bodies, and to proactively support physical health recovery.

In Canada, researchers have identified ways in which treatment programs have integrated physical health interventions such as primary care, obstetrics and gynecology services, dental care, physiotherapy, complementary therapies, and fitness/exercise programs, as part of women’s treatment (15–18). Elsewhere, treatment and recovery programs have also integrated physical health topics into programming, addressing themes such as the effects of substances on women’s health, sexually transmitted and blood borne diseases, nutrition, and women’s health issues overall (19–22). However, it is still not a standard response to offer integrated physical health supports or sex-specific health education in women’s treatment and recovery.

Treatment providers and researchers in our meetings discussed the need to advance both research and practice to better address women’s physical health in recovery. They emphasized the importance of coordinated care, greater access to health care professionals, and the benefits and opportunities of partnering with recreational organizations to integrate physical exercise in therapy. Participants also called for free and trauma-informed dental care, along with training in motivational interviewing and trauma-informed care for medical and dental professionals in order to create a more supportive healthcare environment for women in recovery. In summary, they highlighted the need for increased efforts to improve service integration and the provision and evaluation of physical health interventions.

### 2.2 Responsive to trauma, violence, and mental health concerns

A key issue in women-specific treatment is the high rates of endemic violence against women including intimate partner violence, sexual assault, harassment, stalking, coercive control, and adverse childhood experiences. However, there remains limited programming that addresses violence related and substance use recovery issues together (23). A recent systematic review that mapped strategies for addressing gender-based violence in substance use treatment for women found that risk factors for gender-based violence and addiction included: partner’s substance use, polysubstance use, substance use in sexual relations, lack of social support, financial dependence, childhood sexual abuse, ongoing trauma, recent imprisonment, mental health issues, sex work, and sex with the risk of HIV or pregnancy (24). The systematic review further identified barriers to treatment, such as partner’s substance use, coercive control, fear of child removal, interpersonal stigma, poor communication by health care providers, and a lack of understanding of the mechanisms of violence. Conversely, the most important protective factors included abstinence, family support, and concern for children (24).

While responses to concurrent violence and substance use are much needed, there has been more exhaustive research and practice responses for women experiencing co-occurring mental health and substance use challenges. For example, in our rapid review, a systematic review was identified that described gender-responsive substance use disorder treatment programs for women with co-occurring disorders that suggested that integrated interventions performed similarly, or were more effective, than standard treatment programming, particularly when addressing trauma and mental health issues (21). Integrated interventions such as Seeking Safety (25) and Helping Women Recover (26) showed better outcomes in trauma and psychiatric symptoms, and higher client satisfaction and treatment adherence, compared to standard treatments (21). Several studies also examined the outcomes from a sober living home model that integrates trauma-informed care, gender-responsive and transitional housing, with peer mentoring, gender-based violence support groups, case management, and skills training (27, 28). The findings indicated that women report reduced depression and an increased sense of community, an important factor in women’s recovery.

In the meetings with Canadian treatment providers and researchers, participants described the ways in which they were addressing violence, trauma, and mental health so as to better support women who use substances. Treatment providers noted how they focus on relationship building and creating safe, supportive environments through offering both trauma-informed and trauma-specific services to meet clients’ various needs. Despite these promising practices, they also identified a pressing need for further research into effective prevention and healing intervention strategies to be offered during treatment (29), as well as advanced, accessible trauma-informed training for staff (30). In addition, aftercare interventions that address trauma, violence and substance use concerns in an integrated way (23), and systemic improvements were identified such as those related to housing and poverty that will prevent re-traumatization, support health recovery, and increase stability after treatment.

### 2.3 Supportive of mothers, children, and the mother-child unit

Treatment that is welcoming, accessible, and tailored to pregnant women and gender diverse individuals and new mothers with substance use concerns, and their children is critically needed. Stigma and fear of losing child custody have long acted as very strong barriers to women disclosing substance use and accessing help (31). Treatment programming designed and tailored to meet the needs of mothers *and* children in an integrated way has not been accessible, and instead systems have historically viewed the needs of mothers with substance use concerns and their children as in conflict and disconnected, and therefore not worthy of connected support and treatment (32).

Research is emerging that documents how to provide treatment that effectively meets the needs of pregnant and parenting women with substance use concerns, including codesigned programs (33). Treatment and recovery services that recognize the mother, child, and mother-child bond as three unique foci may be best able to support important attachment and parenting needs, and enhance women’s relational skills and capacity to connect to treatment providers (34, 35). While limited in availability, family centered treatment options, such as those that offer live-in programming for women and their children, often act as a facilitator to women’s treatment access and retention (36, 37). Modalities are essential such as day or outpatient treatment that can ameliorate barriers to care, and integrated, wraparound services that bring together multidisciplinary service providers (38).

Integrated and wraparound programs, including those that offer primary, prenatal and pediatric care; psychiatry; midwifery; counselling; case management; outreach; food and nutritional support; child development and care workers; cultural supports; family supports; peer workers; and housing, employment and income workers have been evidenced in Canada (16, 38–40). Outpatient treatment, community-based services, and modalities such as home visitation and case management have also been found effective to reduce substance use, respond to complex and interconnected social determinants of health, support parenting, and foster the mother-child bond (41–43).

All of these interventions are dependent upon the recognition of the capacity and right of mothers with substance use concerns to parent, and active collaboration between child welfare authorities and substance use treatment systems of care (32, 44).

In our three-part meeting with Canadian treatment providers and researchers, the need for all these approaches were confirmed. The programs that are available echo the research, in that they integrate treatment and comprehensive support. However, while there are some specialized programs dedicated to supporting pregnant and newly parenting women, these are not readily available. Strategies remain very much needed that can facilitate and strengthen linkages between governments and community organizations to support the provision of comprehensive care that addresses maternal health, mental health, child health, substance use, housing, Indigenous-wellness and other wraparound programming elements.

### 2.4 Supportive of connections among women, and individual and collective agency

A final critical area of action in women’s treatment is that which is supportive of women’s empowerment, agency, and connections among women.

Early research from the Women, Co-occurring Disorders and Violence Study (WCDVS) brought together representatives from nine study sites and project consultants to define an empowerment model (45, 46). In doing so, they highlighted that an empowerment model: recognized the partnership between service users and providers, where treatment goals are collaboratively developed; women’s issues are addressed in a wider socioenvironmental context including the pervasiveness of violence and trauma; women’s choices are validated; and women are provided space to support each other, learn from their experiences, and build skills (45). This research was pivotal, as it demonstrated the principles that underly an empowerment model, while also illustrating for the various ways in which empowerment and agency are fostered.

The fostering of connections and community among women has emerged as an important mechanism of empowerment and recovery, with research demonstrating the importance of group cohesion and building a sense of belonging and community (47) - a sentiment also reflected in research on culture-informed approaches to treatment for Indigenous Peoples (8). Peer support models are uniquely positioned to support connections among diverse women and promote empowerment (48–50). Such models provide the context for discussing women’s common “social pain” and inspiring paths to recovery (51, 52).

Empowerment, and the building of networks and support, emerge as key themes in research and practice. Service providers want to be able to offer treatment programs that focus on building self-confidence, assertiveness, and that support women to develop social networks and a more positive self-identity (53, 54). However, more research is needed that considers how experiences of empowerment, connection, and agency are influenced by race/ethnicity, ability, age, income, and other equity factors.

Further, finding measurement tools to accurately reflect women’s experiences and feelings of connection is needed. While qualitative measures, such as client testimonials and journey mapping were highlighted as important for amplifying women’s voices and experiences, quantitative measures were also seen as important for capturing the impact of services in this domain. Documentation of recovery journeys, demonstration of the value of multiple types of support, longitudinal outcomes, and relational measurement tools were all suggested as potential enhancements to current evaluation practices, so that the emphasis on empowerment, connection, and agency continues.

## 3 Discussion

In the late 1990s and early 2000s, advances were made in research and practice on gender responsive substance use treatment for women. In the interim, we have seen increased rates of alcohol and other substance use by women, advances in sex and gender science, attention to social and structural determinants of health, increased understanding of trauma informed care, and emergent wisdom as to Indigenous culture as intervention for addiction, that can all inform sex/gender and equity informed approaches to substance use treatment and recovery. We urgently need a resurgence of interest in gender responsive treatment options that incorporate and reflect the influence of these sex/gender and equity-related factors affecting the health and wellbeing of women who use substances, and their children in Canada and beyond. In a rapid review of evidence and with Canadian service providers, we identified four key areas of women’s treatment that constitute a place to start in making a difference to the lives of women and their children as they navigate substance use treatment and recovery pathways. However, much more research and practice improvement remains to be achieved if we are to address all the sex/gender and equity-related factors influencing substance use by women, the significant barriers to care and recovery, and create overall system improvements that will provide wider access to tailored approaches for a broad range of women and their children.

## Data Availability

The original contributions presented in the study are included in the article/supplementary material. Further inquiries can be directed to the corresponding author.

## References

[1] United Nations Office on Drugs and Crime . Substance Abuse Treatment and Care for Women: Case Studies and Lessons Learned. Vienna, Austria: United Nations Office on Drugs and Crime. (2004).

[2] Canadian Centre on Substance Use and Addiction . Alcohol. (2019). Available online at: https://www.ccsa.ca/sites/default/files/2020-10/CCSA-Canadian-Drug-Summary-Alcohol-2019-en.pdf (Accessed November 11, 2025).

[3] Greaves L Hemsing N . Sex and gender interactions on the use and impact of recreational cannabis. Int J Environ Res Public Health. (2020) 17:509. doi: 10.3390/ijerph17020509., PMID: 31947505 PMC7014129

[4] Keyes KM Jager J Mal-Sarkar T Patrick ME Rutherford C Hasin D . Is there a recent epidemic of women’s drinking? A critical review of national studies. Alcohol Clin Exp Res. (2019) 43:1344–59. doi: 10.1111/acer.14082, PMID: 31074877 PMC6602861

[5] Greaves L Poole N Brabete AC . Sex, gender and alcohol use: Implications for women and low risk drinking guidelines. Int J Environ Res Public Health. (2023) 19:4523. doi: 10.3390/ijerph19084523, PMID: 35457389 PMC9028341

[6] National Institute on Drug Abuse . Sex Differences in Substance Use. National Institute on Drug Abuse website. (2020). Available online at: https://nida.nih.gov/publications/research-reports/substance-use-in-women/sex-differences-in-substance-use (Accessed November 11, 2025).

[7] Rowan M Poole N Shea B Gone JP Myota D Farag M . Cultural interventions to treat addictions in Indigenous populations: Findings from a scoping study. Subst Abuse Treatment Prevention Policy. (2014) 9:1–26. doi: 10.1186/1747-597X-9-34, PMID: 25179797 PMC4158387

[8] Thunderbird Partnership Foundation . Indigenous wellness framework reference guide (2020). Available online at: https://www.thunderbirdpf.org/IWF (Accessed November 11, 2025).

[9] McHugh RK Votaw VR Sugarman DE Greenfield SF . Sex and gender differences in substance use disorders. Clin Psychol Rev. (2018) 66:12–23. doi: 10.1016/j.cpr.2017.10.012, PMID: 29174306 PMC5945349

[10] Substance Abuse and Mental Health Services Administration . Substance Abuse Treatment: Addressing the Specific Needs of Women. Rockville, USA: Treatment Improvement Protocol (TIP) Series No. 51 (2009). 22514859

[11] Brabete AC Greaves L Wolfson L Stinson J Allen S Poole N . Substance use (SU) among women in the context of the corollary pandemics of COVID-19 and Intimate Partner Violence (IPV). (2021). Available online at: https://cewh.ca/wp-content/uploads/2021/04/Rapid-Review-Full-Report-1.pdf (Accessed November 11, 2025).

[12] Greaves L Poole N Brabete AC Hemsing N Stinson J Wolfson L . Integrating Sex and Gender Informed Evidence into Your Practices: Ten Key Questions on Sex, Gender and Substance Use. (2020). Available online at: https://cewh.ca/wp-content/uploads/2022/01/CEWH-02-IGH-Handbook-Web.pdf (Accessed November 11, 2025).

[13] Schmidt R Poole N Greaves L Hemsing N . New Terrain: Tools to integrate trauma and gender informed responses into substance use practice and policy (2018). Available online at: https://cewh.ca/wp-content/uploads/2022/01/NewTerrain_FinalOnlinePDF.pdf (Accessed November 11, 2025).

[14] Centre of Excellence for Women’s Health . Experiences of Women Living with Chronic Pain in Accessing Prescribed Opioids: Scoping Review Executive Summary. (2023). Available online at: https://cewh.ca/wp-content/uploads/2023/04/ChronicPainExecutiveSummary-April24-2023.pdf (Accessed November 11, 2025).

[15] Flannigan K Murphy L Pei J . Integrated supports for women and girls experiencing substance use and complex needs. Subst Abuse: Res Treat. (2023) 17. doi: 10.1177/11782218231208980, PMID: 37954218 PMC10637139

[16] Hubberstey C Rutman D Van Bibber M Poole N . Wraparound programmes for pregnant and parenting women with substance use concerns in Canada: Partnerships are essential. Health Soc Care Community. (2022) 30:e2264–76. doi: 10.1111/hsc.13664, PMID: 34841607

[17] Niccols A Dell CA Clarke S . Treatment issues for Aboriginal mothers with substance use problems and their children. Int J Ment Health Addict. (2010) 8:320–35. doi: 10.1007/s11469-009-9255-8, PMID: 24976814 PMC4071056

[18] Petker T Yanke C Rahman L Whalen L Demaline K Whitelaw K . Naturalistic evaluation of an adjunctive yoga program for women with substance use disorders in inpatient treatment: Within-treatment effects on cravings, self-efficacy, psychiatric symptoms, impulsivity, and mindfulness. Subst Abuse: Res Treat. (2021) 15. doi: 10.1177/11782218211026651, PMID: 34262285 PMC8246483

[19] Ashley OS Marsden ME Brady TM . Effectiveness of substance abuse treatment programming for women: A review. Am J Drug Alcohol Abuse. (2003) 29:19–53. doi: 10.1081/ADA-120018838, PMID: 12731680

[20] Hien DA Campbell AN Ruglass LM Hu MC Killeen T . The role of alcohol misuse in PTSD outcomes for women in community treatment: a secondary analysis of NIDA’s Women and Trauma Study. Drug Alcohol Depend. (2010) 111:114–9. doi: 10.1016/j.drugalcdep.2010.04.011, PMID: 20537811 PMC2981092

[21] Johnstone S Dela Cruz GA Kalb N Tyagi SV Potenza MN George TP . A systematic review of gender-responsive and integrated substance use disorder treatment programs for women with co-occurring disorders. Am J Drug Alcohol Abuse. (2023) 49:21–42. doi: 10.1080/00952990.2022.2130348, PMID: 36283062

[22] Killeen T Hien D Campbell A Brown C Hansen C Jiang H . Adverse events in an integrated trauma-focused intervention for women in community substance abuse treatment. J Subst Abuse Treat. (2008) 35:304–11. doi: 10.1016/j.jsat.2007.12.001, PMID: 18294804 PMC4897772

[23] Brabete AC Greaves L Poole N Wolfson L Whittaker D Cole C . Examining Interventions for Intimate Partner Violence and Substance Use: Results from a Scoping Review. (2024). Available online at: https://cewh.ca/wp-content/uploads/2024/05/Strong-Women-Scoping-Review-Executive-Summary-May-28.pdf (Accessed November 11, 2025).

[24] Romo-Avilés N Tarriño-Concejero L Pavón-Benítez L Marín-Torres J . Addressing gender-based violence in drug addiction treatment: A systematic mapping review. Int J Ment Health Addiction. (2023) 22:3656–82. doi: 10.1007/s11469-023-01072-4

[25] Najavits LM Sullivan TP Schmitz M Weiss RD Lee CSN . Treatment utilization by women with PTSD and substance dependence. Am J On Addict. (2004) 13:215–24. doi: 10.1080/10550490490459889, PMID: 15370941

[26] Covington SS Burke C Keaton S Norcott C Covington SS Burke C . Evaluation of a trauma-informed and gender-responsive intervention for women in drug treatment. J Psychoactive Drugs. (2008) 5:387–98. Available online at: https://search.ebscohost.com/login.aspx?direct=true&AuthType=shib&db=ccm&AN=105530969&site=ehost-live&scope=site&custid=s5672194 (Accessed November 11, 2025)., PMID: 19248396 10.1080/02791072.2008.10400666

[27] Edwards KM Mullet N Siller L . Trauma informed practices of a sober living home for women with addiction and victimization histories. J Soc Work Pract Addict. (2023) 23:102–15. doi: 10.1080/1533256X.2021.2004354

[28] Edwards KM Wheeler L Siller L Murphy SB Ullman SE Harvey R . Outcomes associated with participation in a sober living home for women with histories of domestic and sexual violence victimization and substance use disorders. Traumatology. (2023) 29:191–201. doi: 10.1037/trm0000394

[29] Morton Ninomiya ME Almomani Y Dunbar Winsor K Burns N Harding KD Ropson M . Supporting pregnant and parenting women who use alcohol during pregnancy: A scoping review of trauma-informed approaches. Women’s Health. (2023) 19:17455057221148304. doi: 10.1177/17455057221148304, PMID: 36744547 PMC9905036

[30] Substance Abuse and Mental Health Services Administration . *Addressing the Needs of Women and Girls: Developing Core Competencies for Mental Health and Substance Abuse Professionals* (HHS Pub. No. (SMA) 11-4657). Rockville, USA: Substance Abuse and Mental Health Services Administration (USA). (2011).

[31] Lyall V Wolfson L Reid N Poole N Moritz KM Egert S . The problem is that we hear a bit of everything…”: A qualitative systematic review of factors associated with alcohol use, reduction, and abstinence in pregnancy. Int J Environ Res Public Health. (2021) 18:3445. doi: 10.3390/ijerph18073445, PMID: 33810338 PMC8037183

[32] Schmidt RA Wolfson L Stinson J Poole N Greaves L . Mothering and Opioids: Addressing Stigma and Acting Collaboratively (2019). Available online at: https://cewh.ca/wp-content/uploads/2022/01/CEWH-03-MO-Toolkit_WEB_Update-F-1.pdf (Accessed November 11, 2025).

[33] Bosak J Messersmith L Bryer C Drainoni M Goodman D Adams M . They just looked at me like I was human”: The experiences of parenting women and providers with substance use disorder treatment. J Subst Use Addict Treat. (2024) 157:209240. doi: 10.1016/j.josat.2023.209240, PMID: 38061633

[34] Andrews NCZ Motz M Pepler DJ Jeong JJ Khoury J . Engaging mothers with substance use issues and their children in early intervention: Understanding use of service and outcomes. Child Abuse Negl. (2018) 83:10–20. doi: 10.1016/j.chiabu.2018.06.011, PMID: 29958135

[35] Pepler D Motz M Leslie M Jenkins J Espinet SD Reynolds W . A Focus on Relationships - The Mother Child Study: Evaluating Treatments for Substance-Using Women (2014). Available online at: https://stage.mothercraft.ca/wp-content/uploads/2025/09/The-Mother-Child-Study-FINAL.pdf (Accessed November 11, 2025).

[36] Chou JL Muruthi BM Ibrahim M Janes E Pennington LB Seiler R . A process evaluation of a substance use program for pregnant women: Lessons learned from the field. Int J Ment Health Addict. (2020) 20:455–68. doi: 10.1007/s11469-020-00374-1

[37] Wolfson L Schmidt RA Stinson J Poole N . Examining barriers to harm reduction and child welfare services for pregnant women and mothers who use substances using a stigma action framework. Health Soc Care Community. (2021) 29:589–601. doi: 10.1111/hsc.13335, PMID: 33713525 PMC8251798

[38] Rutman D Hubberstey C . Cross-sectoral collaboration working with perinatal women who use substances: Outcomes and lessons from HerWay Home. J Soc Work Pract Addict. (2020) 20:179–93. doi: 10.1080/1533256X.2020.1793068

[39] Milligan K Tarasoff LA Rodrigues ER Iwajomo T Gomes T de Oliveira C . Neonatal outcomes of pregnant women attending integrated and standard substance use treatment programs in Ontario, Canada. Birth. (2024) 51:284–94. doi: 10.1111/birt.12784, PMID: 37983747

[40] Urbanoski K Joordens C Kolla G Milligan K . Community networks of services for pregnant and parenting women with problematic substance use. PLoS One. (2018) 13:e0206671. doi: 10.1371/journal.pone.0206671, PMID: 30452454 PMC6242306

[41] Graham AV Graham NR Sowell A Ziegler H . Miracle Village: a recovery community for addicted women and their children in public housing. J Subst Abuse Treat. (1997) 14:275–84. doi: 10.1016/S0740-5472(97)00007-X, PMID: 9306303

[42] O’Malley D Chiang DF Siedlik EA Ragon K Dutcher M Templeton O . A promising approach in home visiting to support families affected by maternal substance use. Maternal Child Health J. (2021) 25:42–53. doi: 10.1007/s10995-020-03015-0, PMID: 33245526 PMC7822766

[43] Zweben JE Moses Y Cohen JB Price G Chapman W Lamb J . Enhancing family protective factors in residential treatment for substance use disorders. Child Welfare. (2015) 94:145–66. Available online at: https://search.ebscohost.com/login.aspx?direct=true&AuthType=shib&db=ccm&AN=110870830&site=ehost-live&scope=site&custid=s5672194 (Accessed November 11, 2025)., PMID: 26827469

[44] Drabble L Poole N . Collaboration between addiction treatment and child welfare fields: Opportunities in a Canadian context. J Soc Work Pract Addict. (2011) 11:124–49. doi: 10.1080/1533256X.2011.570657

[45] Elliott DE Bjelajac P Fallot RD Markoff LS Reed BG . Trauma-informed or trauma-denied: principles and implementation of trauma-informed services for women. J Community Psychol. (2005) 33:461–77. doi: 10.1002/jcop.20063

[46] Markoff LS Reed BG Fallot RD Elliott DE Bjelajac P . Implementing trauma-informed alcohol and other drug and mental health services for women: lessons learned in a multisite demonstration project. Am J Orthopsychiatry. (2005) 75:525–39. doi: 10.1037/0002-9432.75.4.525, PMID: 16262512

[47] Valeri L Sugarman DE Reilly ME McHugh RK Fitzmaurice GM Greenfield SF . Group therapy for women with substance use disorders: In-session affiliation predicts women’s substance use treatment outcomes. J Subst Abuse Treat. (2018) 94:60–8. doi: 10.1016/j.jsat.2018.08.008, PMID: 30243419 PMC9976621

[48] Bailey K Trevillion K Gilchrist G . What works for whom and why: A narrative systematic review of interventions for reducing post-traumatic stress disorder and problematic substance use among women with experiences of interpersonal violence. J Subst Abuse Treat. (2019) 99:88–103. doi: 10.1016/j.jsat.2018.12.007, PMID: 30797400

[49] Hubberstey C Rutman D Schmidt R van Bibber M Poole N . Multi-service programs for pregnant and parenting women with substance use concerns: Women’s perspectives on why they seek help and their significant changes. Int J Environ Res Public Health. (2019) 16:3299. doi: 10.3390/ijerph16183299, PMID: 31500358 PMC6765994

[50] SheRecovers . 2024 Outcomes (2025). Available online at: https://sherecovers.org/ (Accessed November 11, 2025).

[51] Najavits LM . Social pain. In: Finding Your Best Self: Recovery form addiction, trauma or both. New York, USA: Guilford (2019). p. 83–7.

[52] Whitaker H . Quit Like a Woman. New York, USA: Penquin Random House (2019).

[53] Bailey K Trevillion K Gilchrist G . ‘We have to put the fire out first before we start rebuilding the house’: Practitioners’ experiences of supporting women with histories of substance use, interpersonal abuse and symptoms of post-traumatic stress disorder. Addict Res Theory. (2020) 28:289–97. doi: 10.1080/16066359.2019.1644323

[54] Schamp J Vanderplasschen W Meulewaeter F . Treatment providers’ perspectives on a gender-responsive approach in alcohol and drug treatment for women in Belgium. Front Psychiatry Front Res Foundation. (2022) 13:941384. doi: 10.3389/fpsyt.2022.941384, PMID: 36111302 PMC9468262

